# 422. Household Economic Burden and Social Consequences of Childhood Severe Pneumonia in India: A Cost of Illness based Pharmacoeconomic Analysis

**DOI:** 10.1093/ofid/ofae631.136

**Published:** 2025-01-29

**Authors:** Maria Tom, Jomel Raju

**Affiliations:** St. Joseph's College of Pharmacy, Cherthala, Kochi, Kerala, India; St. Joseph's College of Pharmacy, Cherthala, Kochi, Kerala, India

## Abstract

**Background:**

Childhood pneumonia stands as a leading cause of mortality among young populations. Providing care for pediatric pneumonia poses significant challenges in resource-constrained regions, where caregivers often bear treatment expenses directly. This study aimed to evaluate the burden, social impact, and economic ramifications of childhood pneumonia, as well as to identify associated risk factors.

**Methods:**

An incidence-based Cost of Illness (COI) study was conducted, focusing on one episode of severe childhood pneumonia from a household perspective. The study was carried out across three distinct sites in the South Indian state of Kerala, India. Through face-to-face interviews, data on socioeconomic status, resource utilization, and associated costs were gathered from caregivers of children aged between 1 month and 6 years. Utilizing a micro-costing bottom-up methodology, medical, non-medical, and time-related costs were computed. Multiple regression analysis was employed to examine factors influencing COI, while sensitivity analysis was conducted to assess the robustness of cost estimations.

**Results:**

A total of 436 children with severe pneumonia were enrolled between January 2022 and December 2023. The mean age of participants was 14 months (SD ±9.1), with males constituting 58.7% (256). The average household cost per episode amounted to USD 149 (95% CI; 139.7 - 155.9), with indirect costs serving as the primary cost drivers (65%, USD 98). While household expenses for the poorest income quintile were lower in absolute terms, they represented a higher proportion of monthly income. Notably, COI was significantly higher for treatments sought at urban health facilities compared to rural counterparts (difference USD 54.3, 95% CI 73.3 to 96.3). Additionally, factors such as age, parental education level, and healthcare facility location emerged as significant predictors of household COI.

**Conclusion:**

This study underscores the substantial economic burden imposed on households by severe childhood pneumonia, with cost differentials evident across various socioeconomic strata. In light of these findings, tailored management strategies should be devised for resource-limited settings to enhance treatment affordability and alleviate household economic strain.

**Disclosures:**

**All Authors**: No reported disclosures

